# Genetic and Biochemical Characterization of 2-Chloro-5-Nitrophenol Degradation in a Newly Isolated Bacterium, *Cupriavidus* sp. Strain CNP-8

**DOI:** 10.3389/fmicb.2017.01778

**Published:** 2017-09-13

**Authors:** Jun Min, Weiwei Chen, Jinpei Wang, Xiaoke Hu

**Affiliations:** ^1^Key Laboratory of Coastal Biology and Bioresource Utilization, Yantai Institute of Coastal Zone Research, Chinese Academy of Sciences Yantai, China; ^2^Key Laboratory of Agricultural and Environmental Microbiology, Wuhan Institute of Virology, Chinese Academy of Sciences Wuhan, China

**Keywords:** 2-chloro-5-nitrophenol, catabolism, *Cupriavidus* sp. strain CNP-8, degradation kinetics, molecular mechanism

## Abstract

Compound 2-chloro-5-nitrophenol (2C5NP) is a typical chlorinated nitroaromatic pollutant. To date, the bacteria with the ability to degrade 2C5NP are rare, and the molecular mechanism of 2C5NP degradation remains unknown. In this study, *Cupriavidus* sp. strain CNP-8 utilizing 2-chloro-5-nitrophenol (2C5NP) and *meta*-nitrophenol (MNP) via partial reductive pathways was isolated from pesticide-contaminated soil. Biodegradation kinetic analysis indicated that 2C5NP degradation by this strain was concentration dependent, with a maximum specific degradation rate of 21.2 ± 2.3 μM h^−1^. Transcriptional analysis showed that the *mnp* genes are up-regulated in both 2C5NP- and MNP-induced strain CNP-8. Two Mnp proteins were purified to homogeneity by Ni-NTA affinity chromatography. In addition to catalyzing the reduction of MNP, MnpA, a NADPH-dependent nitroreductase, also catalyzes the partial reduction of 2C5NP to 2-chloro-5-hydroxylaminophenol via 2-chloro-5-nitrosophenol, which was firstly identified as an intermediate of 2C5NP catabolism. MnpC, an aminohydroquinone dioxygenase, is likely responsible for the ring-cleavage reaction of 2C5NP degradation. Gene knockout and complementation indicated that *mnpA* is necessary for both 2C5NP and MNP catabolism. To our knowledge, strain CNP-8 is the second 2C5NP-utilizing bacterium, and this is the first report of the molecular mechanism of microbial 2C5NP degradation.

## Introduction

Chlorinated nitroaromatic compounds (CNAs) are a group of widely distributed pollutants in the environment throughout the world due to their massive use in the manufacture of herbicides, drugs, dyes and other chemicals (Liu et al., 2011; Tiwari et al., 2017). The natural formation of CNAs is extremely rare. Most of these xenobiotics in the environment are mainly derived from their manufacture, transport and use. Chemical or biological degradation of their derivatives in the environment is another major source. CNAs are highly toxic to humans and animals due to their immunotoxicity, hematotoxicity, teratogenicity and carcinogenicity (Arora et al., 2012a, 2014a). Therefore, many CNAs are listed as priority pollutants by the United State Environmental Protection Agency (USEPA). Recently, removal of these toxicants from the environment has aroused wide concern.

Chloronitrophenols (CNPs) including 2-chloro-4-nitrophenol (2C4NP), 2-chloro-5-nitrophenol (2C5NP), 4-chloro-2-nitrophenol (4C2NP), and 4-chloro-3-nitrophenol (4C3NP) etc are the typical representative of CNAs. They have been detected in industrial effluents and groundwater in many countries, including China. Physico-chemical methods such as photo-fenton process hve been used to remove CNPs from the water; however, this method is cost-consuming and can not degrade CNPs completely (Pradhan et al., 2013). In contrast, biotreatment is considered to be a more cost-effective and thorough strategy to eliminate CNPs from wastewater. Microbial degradation processes of CNPs are particularly being investigated because they can be effectively coupled with the traditional activated sludge process. However, microbial degradation of CNPs is difficult as the simultaneous existence of electron-withdrawing chloro and nitro groups on the aromatic ring makes thses compounds resistant to microbial attack. Therefore, isolation of CNPs-degraders is of great scientific and industrial significance for the detoxification of these toxicants-containing wastewaters.

In addition to bacterial isolation, the knowledge of microbial degradation mechanism of CNPs is also very important, as a comprehensive understanding of microbial catabolic pathway of the target pollutant at biochemical and genetic level would provide valuable insight on understanding the fate of the pollutant in the environments, as well as assessing the population of the functional bacteria during bioremediation (Chi et al., 2013; Wang et al., 2014; Min et al., 2017). So far, several pure microorganisms have been isolated based on their ability to degrade CNPs. *Arthrobacter* sp. SJCon (Arora and Jain, 2011), *Burkholderia* sp. SJ98 (Pandey et al., 2011), *Burkholderia* sp. RKJ 800 (Arora and Jain, 2012a), *Cupriavidus* strain a3 (Tiwari et al., 2017), and *Rhodococcus imtechensis* RKJ 300 (Ghosh et al., 2010) were reported to utilize 2C4NP. Recently, we have revealed the degradation mechanism of 2C4NP in strains SJ98 (Min et al., 2014) and RKJ 300 (Min et al., 2016b). *Exiguobacterium* sp. PMA was reported to utilize 4C2NP as a sole carbon and energy source and degrade 4C2NP with the formation of 4-chloro-2-aminophenol and 2-aminophenol as the intermediates (Arora et al., 2012b). *Pseudomonas* sp. N31 with a plasmid carrying the genes for chlorocatechol degradation was also reported to utilize 4C2NP (Bruhn et al., 1988). In addition, *Bacillus subtilis* RKJ 700 (Arora, 2012), *Pseudomonas* sp. JHN (Arora and Bae, 2014) and *Bacillus* sp. strain MW-1 (Arora and Jain, 2012b) were reported to transform 4C2NP into 5-chloro-2-methyl benzoxazole. Recently, strain JHN was also proved to be able to utilize 4C3NP as a sole carbon and energy source and degrade it with the formation of 4-chlororesorcinol as a metabolite (Arora et al., 2014b).

As another typical representative of CNPs, 2C5NP have also been widely used as intermediates of synthesis of chemical products. Due to its non-volatility and long half-life similar to other nitrophenols (Jaoui et al., 2002; Xiao et al., 2007), 2C5NP is a long-lived pollutant in the environment. The presence of ring-inactivating chloro and nitro groups may makes it recalcitrant to chemical transformation in the natural environment, and microorganisms were proposed to play an more important role in degrading 2C5NP. Although 2C5NP is the structural analog of 2C4NP, 2C5NP is more difficult to be degraded by microorganisms. The aromatics with nitro group at *meta* position are considered to be more resistant to microbial attack than the compounds that have nitro group at *para* position (Arora et al., 2012b). To date, only one bacterium, *Cupriavidus pinatubonensis* JMP134 (formerly *Cupriavidus necator* JMP134 or *Ralstonia eutropha* JMP134), was reported to degrade 2C5NP (Schenzle et al., 1999b), and the molecular mechanism of 2C5NP degradation remains unknown.

In this study, *Cupriavidus* sp. strain CNP-8 was isolated based on its ability to utilize 2C5NP as a sole source of carbon and nitrogen. Biodegradation analysis demonstrated that it is a potential and efficient candidate for biotreatment of 2C5NP-containing industrial effluents. The *mnp* gene cluster involved in the catabolism of 2C5NP was identified from this strain, and the functions of two genes were verified *in vitro* and *in vivo*. Strain CNP-8 isolated here is the second 2C5NP utilizer, and this is the first report of the molecular mechanism of microbial 2C5NP degradation.

## Materials and methods

### Bacteria, plasmids, primers, media, and culture conditions

The plasmids and bacteria are in Table 1, and the primers are in Table S1. *Cupriavidus* strains were grown at 30°C in minimal salt medium (MSM, omitting the CaCl_2_) (Xiao et al., 2006) with different substrates (2C5NP was dissolved in MSM with initial concentration of 5 mM). *E. coli* strains were grown in lysogeny broth (LB) medium at 37°C. When required, chloramphenicol (34 μg/ml), tetracycline hydrochloride (10 μg/ml) and kanamycin (50 μg/ml) was added to the medium. All reagents used here were purchased from Sigma Chemical Co. (St. Louis, MO).

**Table 1:** Bacterial strains and plasmids used in this study.

**Strain or plasmid**	**Relevant genotype or characteristic(s)**	**source**
***Cupriavidus*** **sp**.		
CNP-8	2C5NP and MNP utilizer, wild type	This study
CNP-8Δ*mnpA*	CNP-8 mutant with *mnpA* gene deleted	This study
CNP-8Δ*mnpA*[pRK415-*pnpA*]	*mnpA* gene was complemented by pRK415-*mnpA* in CNP-8Δ*mnpA*	This study
***E**. **coli*** **strains**		
DH5α	*supE44 lacU169 (*φ80*lacZ*ΔM15*) recA1 endA1 hsdR17 thi-1 gyrA96 relA1*	Novagen
Rosetta(DE3)pLysS	F^−^*ompThsdS*(${\text{r}}_{\text{B}}^{-}$${\text{m}}_{\text{B}}^{+}$) *gal dcm, lacY1*(DE3) pLysSRARE (Cm^r^)	Novagen
WM3064	Donor strain for conjugation, 2,6-diaminopimelic acidauxotroph: *thrB1*004 *pro thirpsLhsdS lacZ*ΔM15 RP4-1360 Δ(*araBAD)*567 Δ*dapA*1341::[*ermpir*(wt)]	Lab stock
**PLASMIDS**		
pET-28a	Expression vector, Kan^R^	Novagen
pEX18Tc	Gene knockout vector, *oriT*^+^, *sacB*^+^, Tc^R^	Lab stock
pRK415	Broad host range vector, Tc^R^	Lab stock
pTn*Mod*-Okm	Source of kanamycin resistance gene *nptII*	Lab stock
pET-*mnpA*	*Nde*I-*Xho*I fragment containing *mnpA* cloned into pET-28a	This study
pET-*mnpB*	*Nde*I-*Xho*I fragment containing *mnpB* cloned into pET-28a	This study
pET-*mnpC*	*Nde*I-*Xho*I fragment containing *mnpC* cloned into pET-28a	This study
pET-*mnpD*	*Nde*I-*Xho*I fragment containing *mnpD* cloned into pET-28a	This study
pEX18Tc-*mnpA*	*mnpA* gene knockout vector containing two DNA fragments homologous to the upstream and downstream regions of the *mnpA* and *nptII*	This study
pRK415-*mnpA*	*mnpA* gene complementation vector by cloning *mnpA* into the *Kpn*I/*Eco*RI restriction site of pRK415	This study

### Isolation and characterization of 2C5NP degrader

Strain CNP-8 was isolated from the pesticide-contaminated soil collected from Yantai, China, by enrichment culture method (Liu et al., 2005). This strain was identified by 16S rRNA sequencing with universal primers 27F and 1492R (Polz and Cavanaugh, 1998). Its morphology was observed by scanning electron microscopy (Model S-4800, Hitachi Ltd., Japan).

### 2C5NP degradation studies

Degradation of 2C5NP by strain CNP-8 was investigated by monitoring the OD_600_ and the consumption of substrate. Ammonia concentration was quantified colorimetrically by the Nessler reaction (Krug et al., 1979), and chloride ion was determined by using an ion-selective chloride electrode (Model 96-17, Orion). Degradation of different concentrations of 2C5NP (0.3–0.7 mM) by strain CNP-8 was carried out to study the degradation kinetics. The values of kinetic parameters were derived from modified Gompertz model (Fang et al., 2007; Deng et al., 2016) which could be expressed as follows:

(1) $$\begin{array}{l} S=S_{0}-A^{*}\exp\left\{-\exp\left[\frac{\mu_{\text{m}}}{A}\left(\lambda -t\right)+1\right]\right\} \end{array}$$

*S* is the substrate concentration. *S*_0_ is the initial substrate concentration. *A* is the biodegradation potential. μ_m_ is the maximum biodegradation rate. λ is the lag phase time.

Different carbon source (0.5 and 5 g/L of glucose, succinate or lactate) was added to MSM containing 0.4 mM of 2C5NP to investigate their effect on 2C5NP degradation by strain CNP-8. Biotransformation of *meta*-nitrophenol (MNP) and 2C5NP by strain CNP-8 was carried out as described (Chen et al., 2014). Quantification of 2C5NP and MNP were performed by HPLC analysis. In order to identify the intermediates of 2C5NP degraded by strain CNP-8, 2C5NP was incubated under anaerobic condition with 2C5NP-induced cells, and the metabolites were identified by GC-MS analysis.

### Analytical methods

HPLC assay was performed as previously described (Min et al., 2016a). MNP and 2C5NP were quantified at 280. The authentic MNP and 2C5NP had retention times of 10.5 and 13.4 min, respectively. The HPLC-MS condition was the same as described previously (Min et al., 2016a) except the gradient mobile phase was different. The mobile phase consisted of A (acetonitrile) and B (H_2_O) with the following gradients: 0–5 min, 5% A; 5–10 min, 5–20% A; 10–20 min, 20–40% A; 20–25 min, 40% A. The condition of GC-MS analysis was the same as described previously (Zhang J. J. et al., 2009).

### Real-time quantitative PCR, gene cloning, protein expression and purification

Whole-genome sequencing and annotation of strain CNP-8 was performed in the Novogene Bioinformatics Institute (Beijing, China). The transcriptional levels of *mnp* genes were performed by real-time quantitative PCR (RT-qPCR) as described (Min et al., 2016a) with primers described in Table S1. The relative expression levels of target genes were calculated according to the $2^{-\Delta \Delta C_{T}}$ method with the 16S rRNA as a reference gene (Livak and Schmittgen, 2001). *mnpA, mnpB, mnpC*, and *mnpD* genes amplified from the genomic DNA (the primers are listed in Table S1), were respectively cloned into pET-28a to obtain the expression plasmids (Table 1). The expression plasmids were subsequently introduced into *E. coli* Rosetta(DE3)pLysS, and the protein expression and purification was performed as previously described (Liu and Zhou, 2012).

### Enzymatic assays

The catalytic activity of MnpA against 2C5NP and MNP was analyzed in the reaction mixture (1 ml) containing, 0.2 mM NADPH, 0.1–5 μg of purified H_6_-MnpA, 50 μM substrate (2C5NP or MNP) and 50 mM phosphate buffer (pH 7.5). The reference cuvette contained all components except the substrate, and the activity assay was initiated with the addition of substrate. During identification of products, the experiment was performed under anaerobic conditions to prevent autoxidation of the products. The reaction mixture was extracted with ethyl acetate after HCl acidification, and the ethyl acetate phase was then collected and dried using anhydrous sodium sulfate before HPLC-MS analysis. In the kinetics assays, three independent experiments were carried out with 7 substrate concentrations (2C5NP: 5–50 μM; MNP: 2–20 μM), while the concentration of NADPH was fixed at 200 μM. The Michaelis-Menten equation was fitted to the data by using OriginPro 8. Bradford method (Bradford, 1976) was used to determine the protein concentration. One unit of enzyme activity was defined as the amount of enzyme consuming 1 μmol of substrate (2C5NP or MNP) in 1 min. The dioxygenase activity of MnpC toward hydroquinone and chlorohydroquinone was measured as described for aminohydroquinone dioxygenase (Yin and Zhou, 2010).

### Gene knockout and complementation of *mnpA*

Gene knockout and complementation were performed as previously described (Min et al., 2014). Plasmid pEX18Tc-*mnpA* for *mnpA* knockout was constructed by fusing kanamycin resistance gene (*nptII*) and the upstream and downstream fragments of *mnpA* to *Eco*RI/*Hin*dIII digested pEX18Tc by using in-fusion HD cloning kit (Takara) (the primers are listed in Table S1, and the plasmids are in Table 1). Plasmid pRK415-*mnpA* used for *mnpA* complementation was obtained by cloning *mnpA* to the *Hin*dIII/*Kpn*I digested pRK415. The ability of strains CNP-8Δ*mnpA* and CNP-8Δ*mnpA*[pRK415-*mnpA*] to utilize substrate (2C5NP or MNP) was examined by determining bacterial growth and substrate consumption.

### Nucleotide sequence accession numbers

The GenBank accession numbers for the nucleotide sequence of 16S rRNA, *mnp* gene cluster and *mnpB* gene reported in this paper are KY643479, KY937901, and KY937902, respectively.

## Results and discussion

### Isolation and classification of strain CNP-8

**Figure 1:**
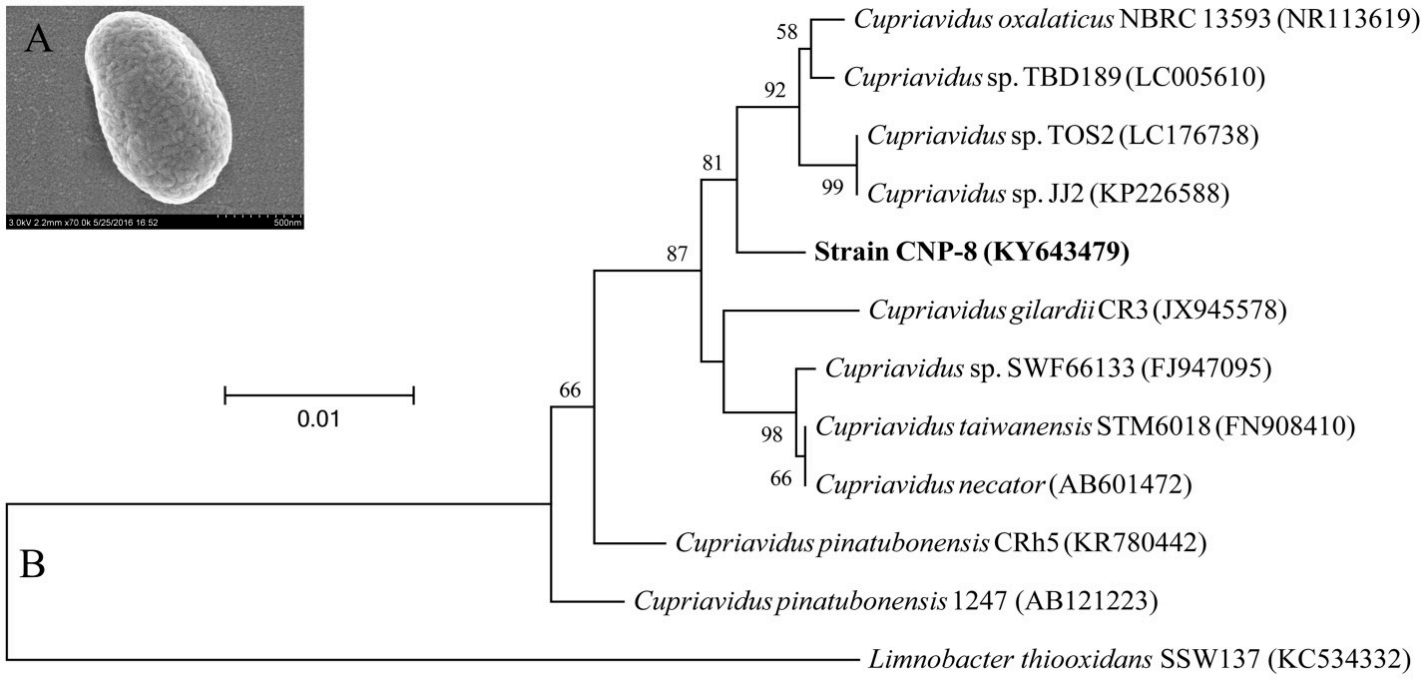
Identification of the newly isolated *Cupriavidus* sp. strain CNP-8. **(A)** The morphological observation of strain CNP-8 by scanning electron microscopy. **(B)** The phylogenetic tree of strain CNP-8 based on 16S rRNA gene sequence analysis. Limnobacter thiooxidans SSW137 was used as outgroup. GenBank accession numbers are shown in parentheses.

A 2C5NP-degrading bacterium, designated strain CNP-8, was isolated from pesticide-contaminated soil with 2C5NP as a sole source of carbon and nitrogen. It was characterized as Gram-negative by Gram staining, and observed as short-rod-shaped by scanning electron microscopy (Figure 1A). It was identified as a member of *Cupriavidus* based on 16S rRNA sequence analysis. The phylogenetic relationships of the 16S rRNA gene sequences of strain CNP-8 and other representative *Cupriavidus* strains are shown in Figure 1B. Before this study, there is only one bacterium, *Cupriavidus pinatubonensis* JMP134 (Schenzle et al., 1999b), was reported to be able to degrade 2C5NP.

### Biodegradation of 2C5NP by strain CNP-8

#### Strain CNP-8 degrades 2C5NP with release of ammonium and chloride ion

**Figure 2:**
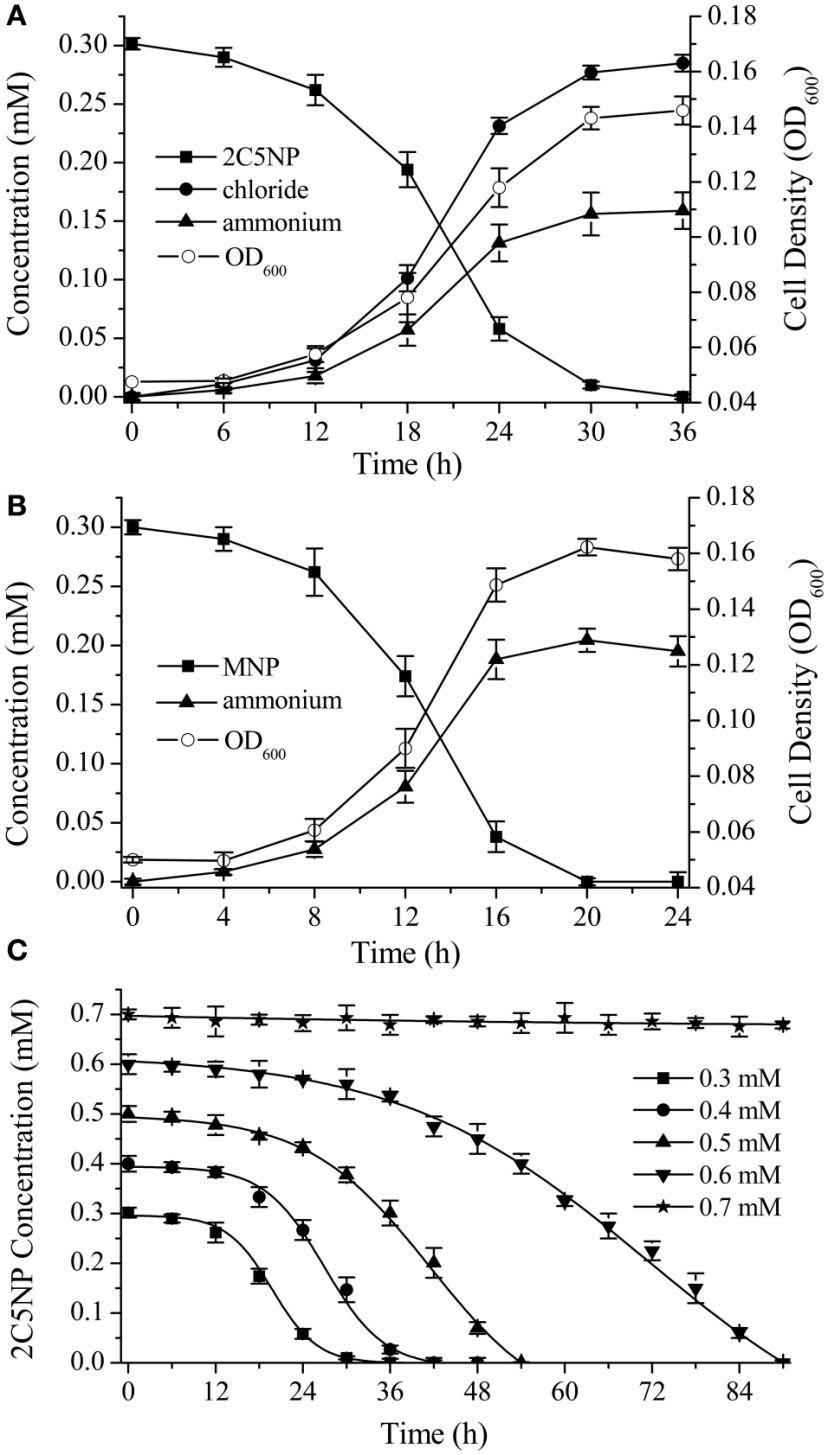
Biodegradation of 2C5NP **(A)** and MNP **(B)** by *Cupriavidus* sp. strain CNP-8, together with the accumulation of chloride, ammonia and the bacterial biomass (indicated by OD_600_). **(C)** Effect of different 2C5NP concentrations on 2C5NP biodegradation by strain CNP-8. All experiments were performed in triplicate with initial OD_600_ of approximately 0.05. Error bars indicate standard deviations.

Strain CNP-8 degraded 0.3 mM of 2C5NP completely after 36 h of incubation with release of ammonium and chloride ion (Figure 2A). Meanwhile, the biomass increased apparently, with OD_600_ increasing from initial 0.048 to final 0.146. This clearly revealed that strain CNP-8 is capable to utilize 2C5NP as sole sources of carbon and nitrogen, and the cell growth was closely correlated with the amount of substrate utilized. In particular, the cell growth exhibited a lag phase of about 12 h, and then increased rapidly for the rest of the incubation period, indicating that strain CNP-8 had an induction period prior to utilize 2C5NP. In addition to 2C5NP, strain CNP-8 was also found to be able to utilize MNP with the release of ammonium, and the OD_600_ increased from 0.051 to 0.162 within 20 h (Figure 2B). The higher degradation rate of MNP than 2C5NP is apparently due to that 2C5NP is much more recalcitrant and toxic as compared to MNP. As ammonium was released during the catabolism of both 2C5NP and MNP, strain CNP-8 was assumed to degrade these two nitrophenols via the partial reductive pathways.

#### 2C5NP degradation kinetics

Biodegradation of 2C5NP at different concentrations was carried out to determine the kinetic parameters of 2C5NP degradation by strain CNP-8. This knowledge is important in understanding the capability of the degrader and designing the bioremediation process (Shen et al., 2009). Because the growth of strain CNP-8 was coupled to the 2C5NP consumption, the substrate biodegradation can be quantitatively characterized using a modified Gompertz model (Fang et al., 2007; Deng et al., 2016). As shown in Figure 2C, strain CNP-8 degraded 0.3 mM of 2C5NP completely in 36 h, followed by 0.4 mM in 42 h, 0.5 mM in 54 h, and 0.6 mM in 90 h. Accelerated biodegradation of 2C5NP was described well by the modified Gompertz model, with R^2^ more than 98.8% (Table 2). The biodegradation lag phase was prolonged apparently with the increase of 2C5NP concentration, and the maximum biodegradation rate was 21.2 ± 2.3 μM h^−1^ when the substrate concentration was 0.4 mM. However, negligible degradation of 2C5NP was observed when the substrate exceeded 0.7 mM. This indicated that 2C5NP at high concentrations exhibited toxic inhibitory effect on growth of strain CNP-8. Indeed, microbial degradation of chloronitrophenols such as 2C4NP (Arora and Jain, 2012a; Tiwari et al., 2017) and 4C2NP (Arora et al., 2012b), as the isomers of 2C5NP, has been proved to be concentration dependent.

**Table 2:** Kinetic parameters of 2C5NP degradation by strains CNP-8.

**Strains**	**Initial 2C5NP (μM)**	**λ (h)^a^**	**emphμ_m_(μM h^−1^)^b^**	***R*^2^**
CNP-8	302	12.1 ± 0.8	20.0 ± 1.6	0.988
	409	16.3 ± 1.3	21.2 ± 2.3	0.988
	504	24.9 ± 1.0	17.7 ± 1.6	0.995
	610	32.9 ± 1.8	11.9 ± 1.1	0.991
CNP-8Δ*mnpA*[pRK415-*mnpA*]	404	15.5 ± 1.7	14.7 ± 0.8	0.989

*Minimal salt medium (MSM) with different concentrations of 2C5NP was inoculated by strain CNP-8 with initial OD_600_ of 0.05, and shaken at 30°C and 180 rpm*.

a *λ is the lag phase time*.

b *μ_m_ is the maximum biodegradation rate*.

#### Effect of supplemented carbon on 2C5NP degradation

Different concentrations of glucose, succinate or lactate were supplemented to MSM with 0.4 mM of 2C5NP to investigate the effect of additional carbon sources on the degradation of 2C5NP by strain CNP-8. This is of practical importance for biotreatment of 2C5NP-containing industrial wastewater or soil because several reports have shown that nutrient supplement can stimulate growth of pollutant degraders and enhance their ability to degrade contaminants (Cheung and Kinkle, 2005; Zhong et al., 2007), while some other studies have claimed that changes in nutrients could produce negative effects (Carmichael and Pfaender, 1997; dos Santos et al., 2009). In this study, addition of all nutrients with concentration of 0.5 g/L enhanced the degradation of 2C5NP, and succinate addition exhibited maximum degradation rates (Table 3). However, the 2C5NP degradation was found to be inhibited when the concentration of nutrient was increased up to 5 g/L, although the biomass increased remarkably. This indicated that supplemental nutrient could enhance 2C5NP degradation as a result of increase in biomass, but in a dose dependent manner. This is similar with the report of *para*-nitrophenol degradation by *Rhodococcus* sp. strain CN6 (Zhang J. et al., 2009). A more interesting finding is that strain CNP-8 was found to be able to remove 0.7 mM of 2C5NP in 90 h when 0.5 g/L of succinate was added, although it is unable to grow on MSM containing only the same amount of substrate. We speculate that the simultaneous utilization of supplemental nutrients and 2C5NP enables strain CNP-8 to mitigate the 2C5NP toxicity by available nutrient and consequently by the build-up of more biomass. This hypothesis has been proposed previously to explain the degradation of phenol (dos Santos et al., 2009). Herein, the degradation of 2C5NP by strain CNP-8 was investigated in liquid MSM whose composition was similar with some reported synthetic wastewater (Ahmadi et al., 2015; Tiwari et al., 2017); therefore, the result in this study is likely able to extrapolate to the real wastewater.

**Table 3:** Effect of different carbons on degradation of 2C5NP by strain CNP-8.

**Carbon source**	**Concentration (g/L)**	**OD_600_^a^**	**Time required for complete degradation of 2C5NP (h)**	**Degradation rate (μM h^−1^)^b^**
Control	-	0.171 ± 0.012	42.4 ± 2.5	9.43
glucose	0.5	0.332 ± 0.026	24.7 ± 1.6	16.19
	5	1.587 ± 0.035	60.1 ± 2.4	6.65
succinate	0.5	0.354 ± 0.018	22.5 ± 2.1	17.78
	5	1.632 ± 0.027	53.3 ± 3.8	7.50
lactate	0.5	0.312 ± 0.032	26.5 ± 1.4	15.09
	5	1.534 ± 0.024	63.4 ± 3.5	6.31

*Minimal salt medium (MSM) with 0.4 mM of 2C5NP was inoculated by strain CNP-8 with initial OD_600_ of 0.05, and shaken at 30°C and 180 rpm. Control: without added carbon source*.

a *OD_600_ was determined at the time of complete degradation of 2C5NP*.

b *Rate of 2C5NP degradation (μM h^−1^) = 400/time required for complete degradation of 2C5NP*.

### 2C5NP degradation is induced by either 2c5np or MNP

**Figure 3:**
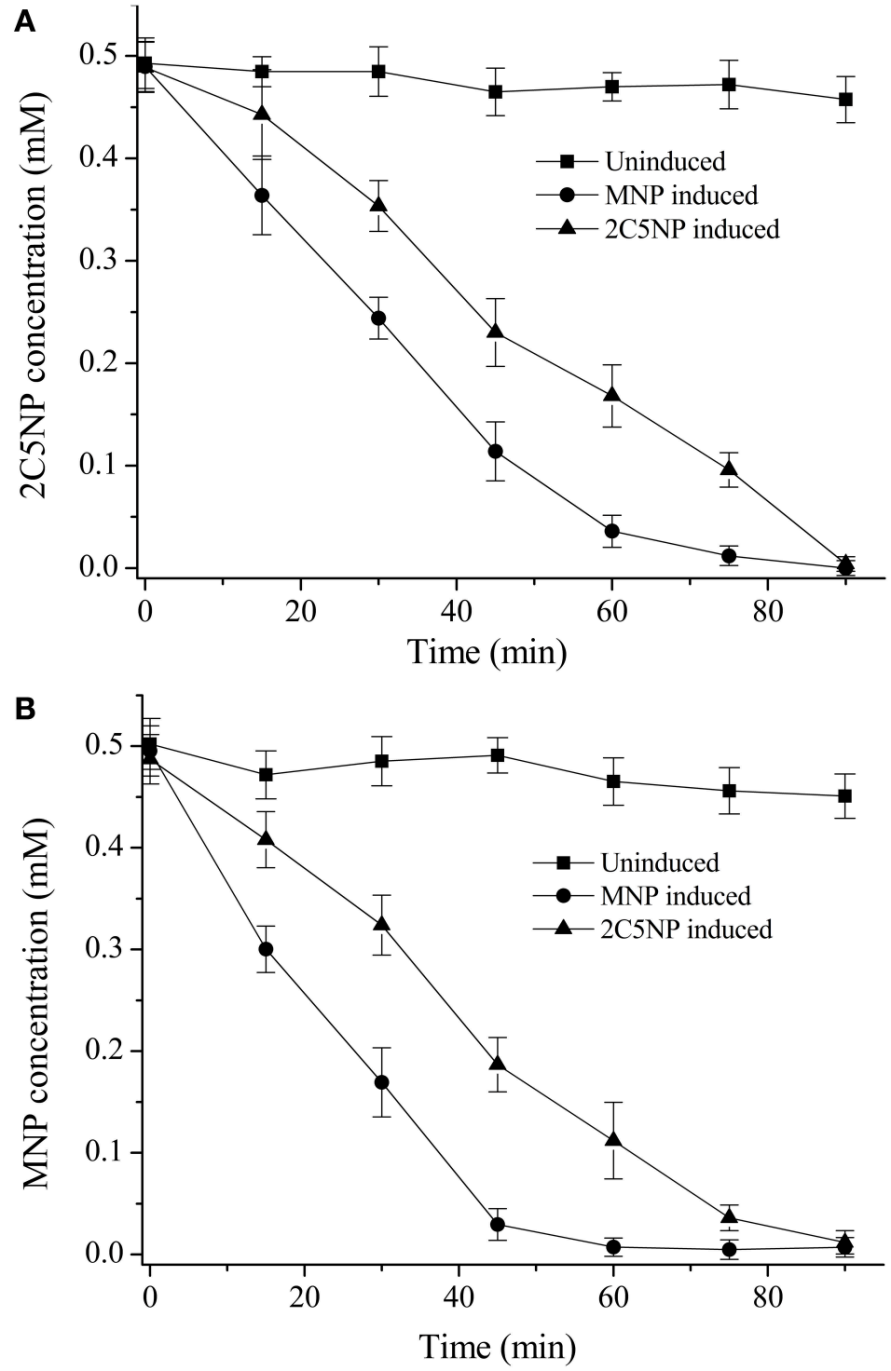
Biotransformation of 2C5NP **(A)** and MNP **(B)** by the induced and uninduced cells of *Cupriavidus* sp. strain CNP-8. The cells were suspended to OD_600_ of 1.0 with phosphate buffer, and 0.5 mM substrate was added. All experiments were performed in triplicate, and error bars indicate standard deviations.

Considering that strain CNP-8 degraded both MNP and 2C5NP via partial reductive pathways; therefore, biotransformation analysis was initially carried out to determine whether the metabolism of 2C5NP and MNP share the enzymes. The un-induced strain CNP-8 had no activity for both 2C5NP and MNP (Figures 3A,B), whereas the 2C5NP-induced cells degraded 2C5NP with a rate of 5.22 ± 0.47 μM/OD_600_ of cell/min and MNP-induced cells degraded MNP with a rate of 10.34 ± 1.21 μM/OD_600_ of cell/min, indicating that the genes responsible for 2C5NP and MNP degradation in strain CNP-8 were inducible. Furthermore, MNP-induced cells can degrade 2C5NP (8.35 ± 0.87 μM/OD_600_ of cell/min), while 2C5NP-induced strain CNP-8 was found to be able to transform MNP (6.01 ± 0.54 μM/OD_600_ of cell/min). This suggests that the enzymes involved in 2C5NP degradation are likely also responsible for the catabolism of MNP in strain CNP-8. No intermediate was detected when strain CNP-8 was grown in MSM with 2C5NP as sole sources of carbon and nitrogen. However, a metabolite was captured by GC-MS analysis when 2C5NP was incubated under anaerobic condition with 2C5NP-induced cells. This intermediate was proposed as aminohydroquinone based on the molecular ion peak at m/z 125 (Figure S1).

### Sequence analyses of 2C5NP catabolic genes

**Figure 4:**
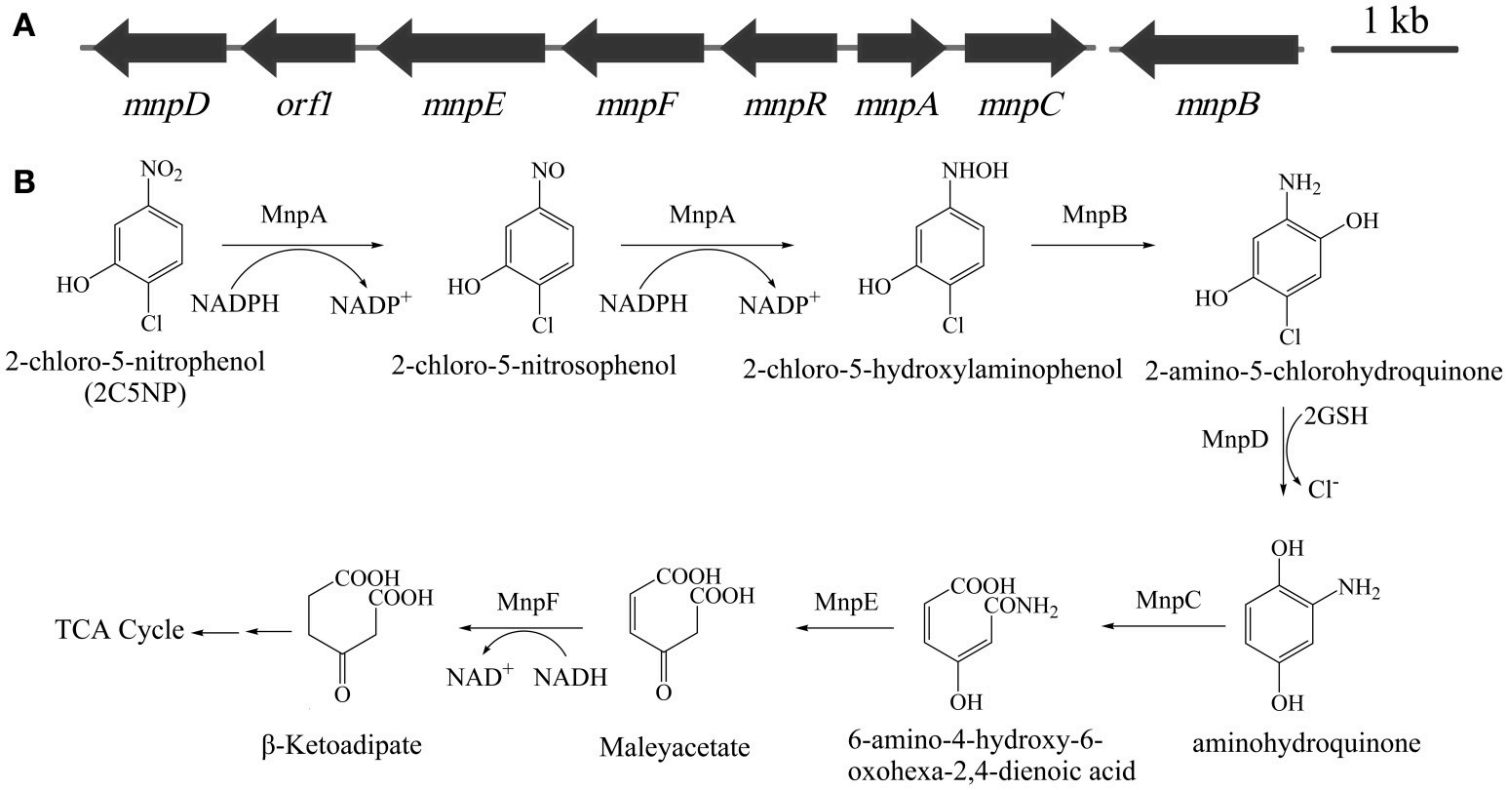
**(A)** Organization of the *mnp* gene cluster of *Cupriavidus* sp. strain CNP-8. The black arrows indicate the sizes and directions of transcription of each gene. **(B)** Proposed pathway for 2C5NP catabolism in strain CNP-8, together with the catabolic reactions catalyzed by *mnp* gene products.

The above finding revealed that metabolism of 2C5NP and MNP in strain CNP-8 may share identical enzymes, which motivate us to identify their coding genes. This knowledge is very significant because the molecular mechanism of microbial 2C5NP degradation remains unknown. Initially, the draft genome of strain CNP-8 was sequenced. A gene cluster designated as *mnp* (Figure 4A) was identified from the contig 80 by comparative genomics analysis with *Cupriavidus pinatubonensis* JMP134. The proteins encoded by *mnpA* and *mnpC* exhibit high homology with MNP nitroreductase (93% identity) (Yin et al., 2010) and aminohydroquinone dioxygenase (94% identity) (Yin and Zhou, 2010), respectively, which were reported to be involved in MNP catabolism in strain JMP134. *mnpD* appears to encode a reductive dechlorinase belong to the glutathione S-transferase family as the level of identity of MnpD with the 2,5-dichlorohydroquinone reductive dehalogenase from *Sphingomonas paucimobilis* UT26 is 40% (Kumari et al., 2002). MnpR, MnpE, and MnpF were proposed to be LysR regulatory protein, amidase and maleylacetate reductase, respectively, by BLAST analysis with the available genome sequence of strain JMP134. In addition, a gene (designed as *mnpB*) encoding a protein with 99% of identity with the 3-hydroxylaminophenol mutase involved in MNP catabolism in strain JMP134 (Schenzle et al., 1999a) was identified from the contig 35 by comparative genome analysis.

### *mnp* genes are up-regulated in 2C5NP-induced strain CNP-8

**Figure 5:**
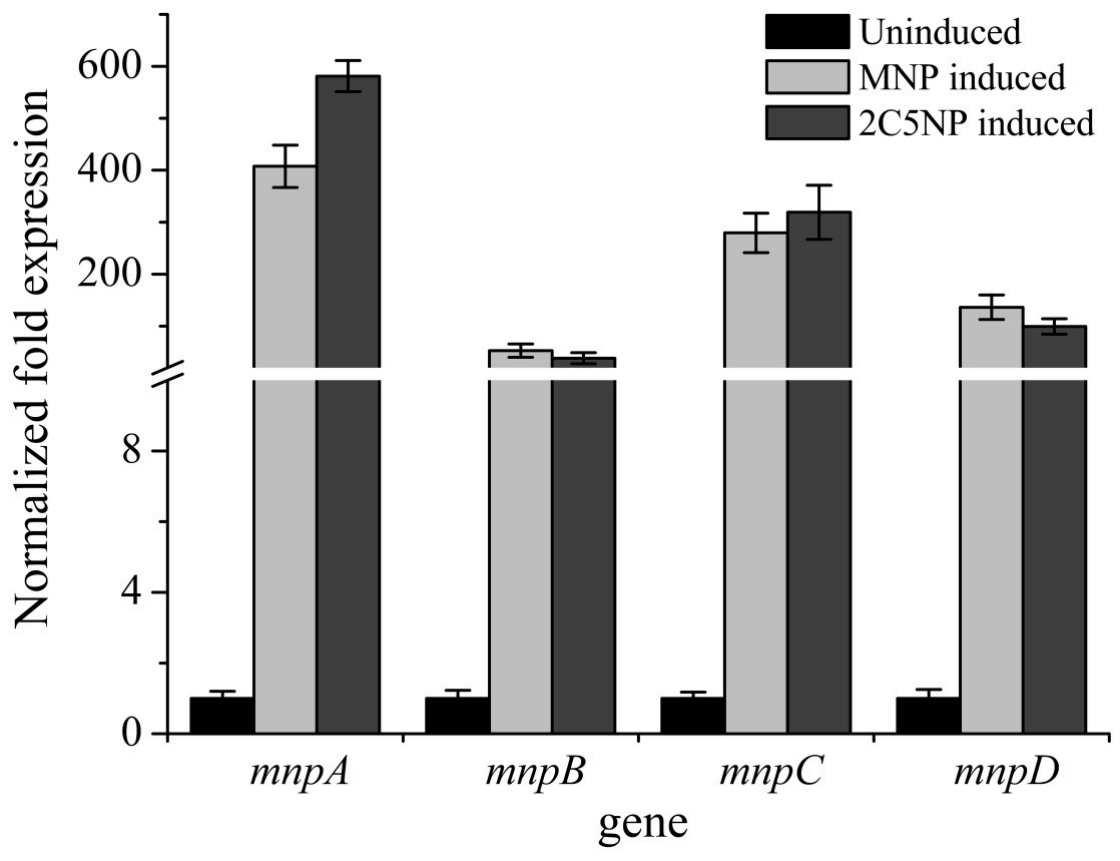
Transcriptional analyses of the representative *mnp* genes in strain CNP-8 under induced and uninduced conditions by RT-qPCR. All experiments were performed in triplicate, and error bars indicate standard deviations.

RT-qPCR showed that the transcription levels of *mnpA, mnpB, mnpC*, and *mnpD* under 2C5NP-induced condition were significantly increased in comparison with the un-induced condition (Figure 5), with 518-, 38-, 319-, and 99-fold increase, respectively. The differences of transcription level among *mnpAC, mnpB* and *mnpD* is likely due to that they were located on different operons and transcribed independently. Similarly, the *mnp* genes are also up-regulated under MNP-induced condition, with 407-, 53-, 279-, and 136-fold increase, respectively. This finding indicated that the *mnp* genes are likely responsible for both 2C5NP and MNP catabolism in strain CNP-8.

### Expression and purification of mnp proteins

A total of 34 mg recombinant MnpA with specific activity of 33.6 U mg^−1^ for 2C5NP (47.5 U mg^−1^ for MNP) was purified from 500 ml of culture. Purified fractions of H_6_-MnpA were yellow, and the enzyme has absorption peaks at 370 and 450 nm, consistent with previously reported flavoprotein nitroreductases (Somerville et al., 1995; Yin et al., 2010). For H_6_-MnpB purification, 25.4 mg enzyme was obtained from 500 ml of culture. Although a certain amount of MnpC was inclusion body, 4.2 mg of H_6_-MnpC was purified from 3,000 ml of culture. Unfortunately, only a very small amount of H_6_-MnpD was soluble, and extremely little H_6_-MnpD was purified even 9,000 ml of culture was collected. SDS-PAGE analysis of the purified Mnp proteins showed that the molecular masses of H_6_-MnpA, H_6_-MnpB and H_6_-MnpC are about 26, 52, and 35 kDa (Figure S2), respectively, consistent with their deduced molecular masses.

### MnpA catalyzes the partial reduction of 2C5NP to 2-chloro-5-hydroxylaminophenol via 2-chloro-5-nitrosophenol

**Figure 6:**
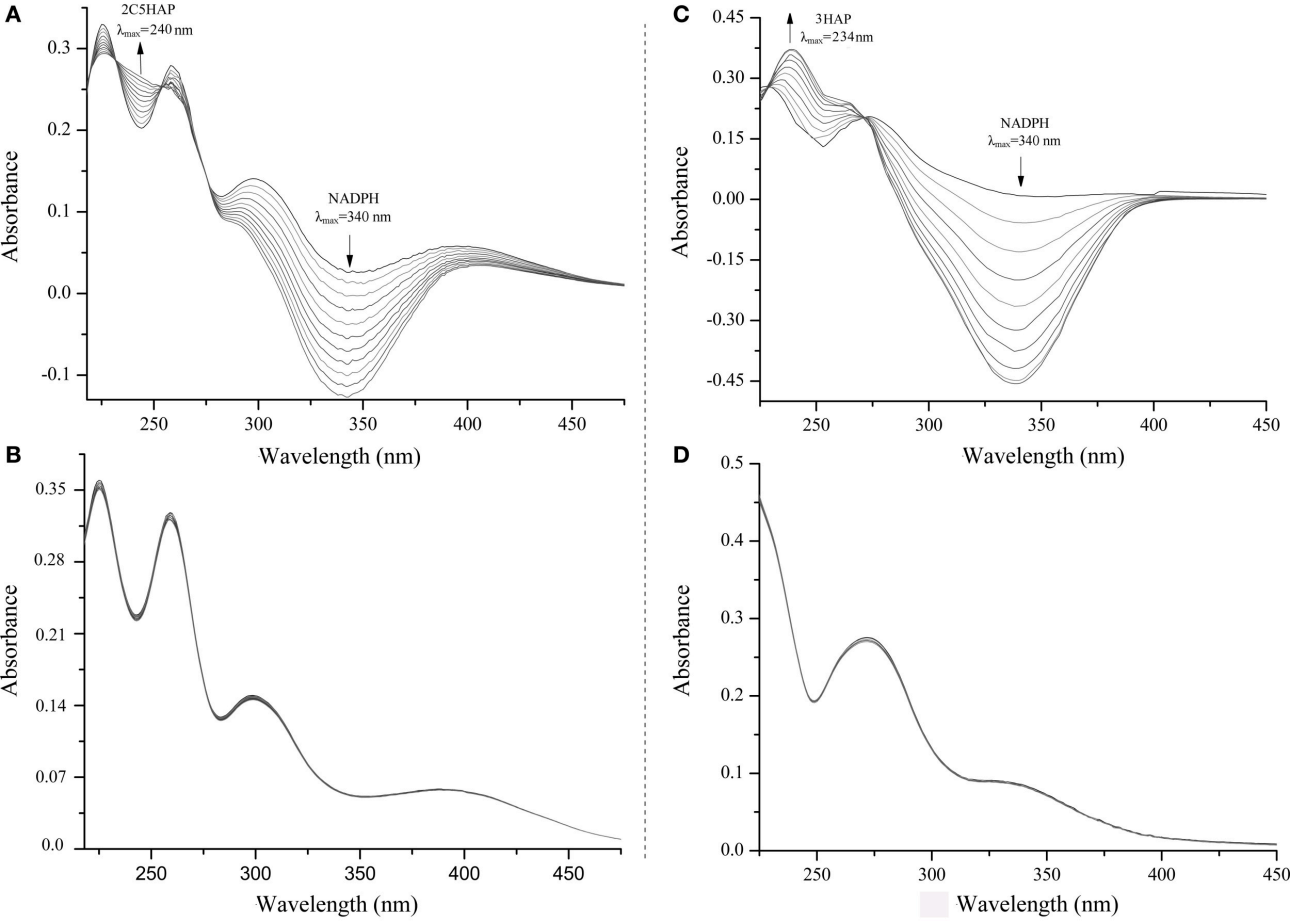
Spectral changes during the transformation of 2C5NP (**A**: with H_6_-PnpA; **B**: without H_6_-PnpA) and MNP (**C**: with H_6_-PnpA; **D**: without H_6_-PnpA). The spectra were recorded every minute after the addition of substrate. The arrows indicate the directions of spectral changes.

*E. coli* Rosetta(DE3) carrying pET-*mnpA* had the ability to degrade 2C5NP and MNP by HPLC analysis, whereas neither 2C5NP nor MNP consumption was detected when the *E. coli* cells only containing plasmid pET-28a. Furthermore, the purified H_6_-MnpA transformed MNP rapidly, together with the consumption of NADPH (λ_max_ = 340 nm) and accumulation of a metabolite with a λ_max_ of 234 nm (Figure 6C), in accord with the spectral property of 3-hydroxylaminophenol as reported (Schenzle et al., 1999b). Previously, the MNP nitroreductase from *Cupriavidus pinatubonensis* JMP134 have been reported to catalyze the reduction of MNP (Yin et al., 2010), but its catalytic activity for 2C5NP has not been characterized. In this study, H_6_-MnpA was found to be able to catalyze the degradation of 2C5NP, together with consumption of NADPH (Figure 6A). Two isobestic points at 232 and 254 nm, respectively, were observed, suggesting the conversion of 2C5NP to a new product (λ_max_ ≈240 nm). In contrast, no spectral change occurred when His_6_-MnpA was omitted from the reaction mixtures (Figures 6B,D).

**Figure 7:**
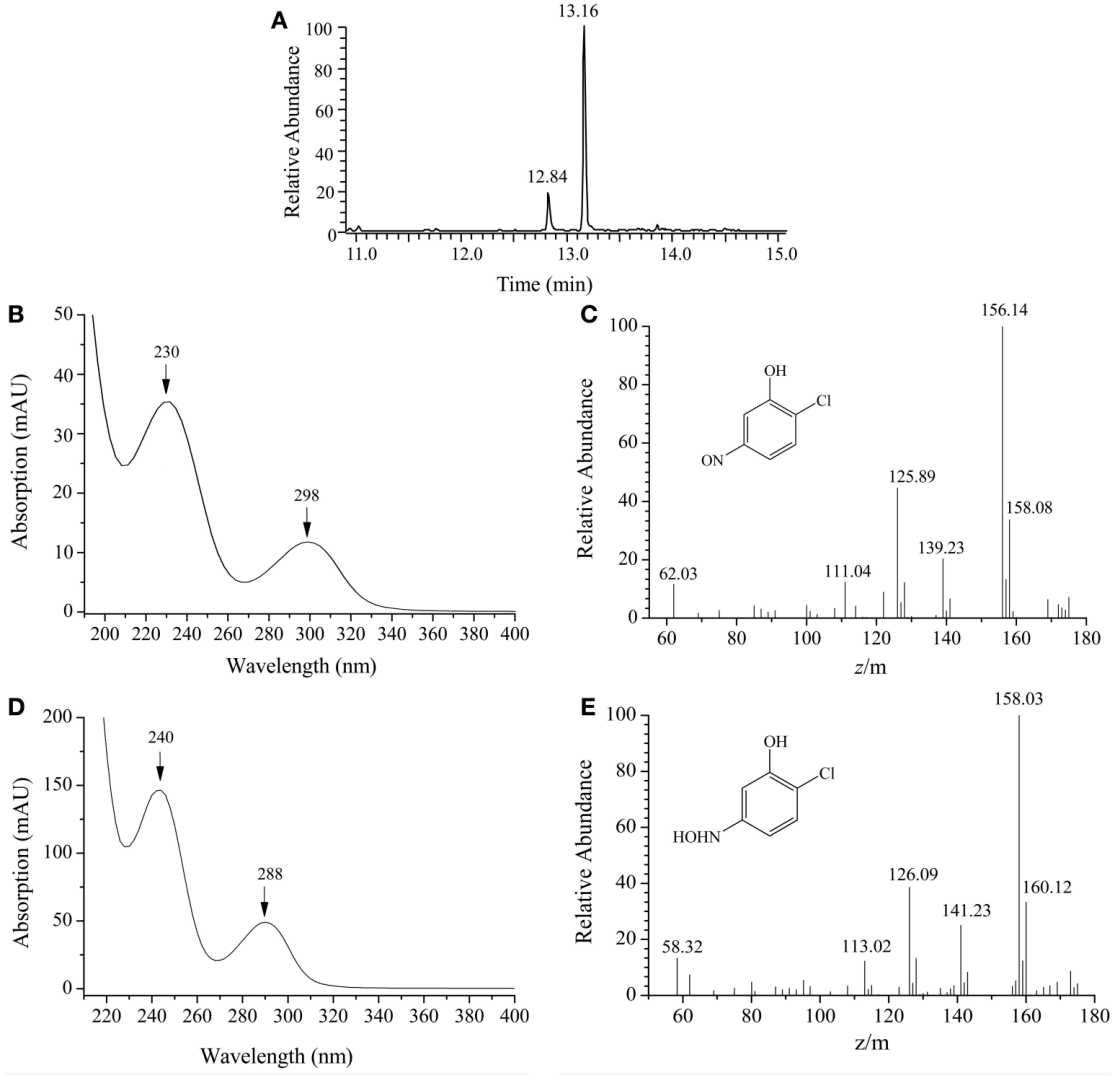
HPLC-MS analysis of the products captured during 2C5NP conversion by purified H_6_-PnpA. **(A)** HPLC chromatogram of the products; **(B)** photospectrometry of 2-chloro-5-nitrosophenol; **(C)** mass spectrometry of 2-chloro-5-nitrosophenol; **(D)** photospectrometry of 2-chloro-5-hydroxylaminophenol; **(E)** mass spectrometry of 2-chloro-5-hydroxylaminophenol.

By HPLC-MS analysis, two products with retention times of 12.84 and 13.16 min, respectively, were detected when 2C5NP was transformed by purified H_6_-MnpA under anaerobic conditions (Figure 7A). Metabolite A (λ_max_ = 230, 298 nm) was suggested as 2-chloro-5-nitrosophenol, with a deprotonated ion at *m/z* 156.14 and its fragments at *m/z* 125.89 (loss of −NO) and at *m/z* 139.23 (loss of −HO) (Figures 7B,C). Metabolite B (λ_max_ = 240, 288 nm) has the same spectral property with synthetic 2-chloro-5-hydroxylaminophenol (Schenzle et al., 1999b), which has a deprotonated ion at *m/z* 158.03 and the fragments at *m/z* 126.09 (loss of −NHOH) and at *m/z* 141.23 (loss of −HO) (Figures 7D,E). On the basis of the products identification of MnpA, strain CNP-8 was further proved to degrade 2C5NP via a partial reductive pathway (Figure 4B), apart from the release of ammonium during 2C5NP degradation. The partialy-purified nitroreductase from *Cupriavidus pinatubonensis* JMP134 was previously proved to catalyze the transformation of 2C5NP to 2-chloro-5-hydroxylaminophenol (Schenzle et al., 1999b), but its protein sequence and coding gene were not reported. Moreover, 2-chloro-5-nitrosophenol, the initial intermediate of microbial 2C5NP degradation, was detected for the first time in this study.

Neglectable activity of H_6_-MnpA for both 2C5NP and MNP was observed when NADPH in the reaction mixture was substituted by NADH, indicating that MnpA prefers NADPH than NADH as the co-substrate, consistent with other reported nitroaromatic nitroreductases (Somerville et al., 1995; Yin et al., 2010). Enzymatic kinetics assays revealed that H_6_-MnpA exhibit a higher affinity and catalytic efficiency for MNP (*K*_m_ = 3.4 ± 1.63 μM, *k*_cat_/*K*_m_ = 342 ± 47.6 μM^−1^ min^−1^) than 2C5NP (*K*_m_ = 16.3 ± 2.87 μM, *k*_cat_/*K*_m_ = 158 ± 31.2 μM^−1^ min^−1^), suggested that MNP is the physiological substrate for MnpA in strain CNP-8.

### MnpB, PnpC and MnpD are likely involved in 2C5NP catabolism in strain CNP-8

The enzymatic analysis of MnpB was not carried out in this study since the commercial standard of its substrate is unavailable. However, considering that MnpB exhibits extremely high degree of identity with the 3-hydroxylaminophenol mutase (accession number WP_011298219), which was capable to catalyze the transformation of synthetic 2-chloro-5-hydroxylaminophenol to 2-amino-5-chlorohydroquinone (Schenzle et al., 1999a), and its coding gene is highly transcribed in 2C5NP-induced strain CNP-8; therefore, it is reasonable to conclude that MnpB is responsible for transformation of 2-chloro-5-hydroxylaminophenol to 2-amino-5-chlorohydroquinone during 2C5NP degradation by strain CNP-8 (Figure 4B). Purification of H_6_-MnpD failed, which hampered its enzymatic analysis *in vitro*. However, MnpD shares moderate identity (40%) with LinD from *Sphingomonas paucimobilis* UT26 which was reported to catalyze dechlorination of 2,5-dichlorohydroquinone, a structure analog of 2-amino-5-chlorohydroquinone, to 2-chlorohydroquinone (Kumari et al., 2002). Moreover, *mnpD*, located upstream of the 2C5NP nitroreductase-encoding *mnpA*, is up-regulated in 2C5NP-induced strain CNP-8. These combined data suggested that MnpD is lilely responsible for dechlorination of 2-amino-5-chlorohydroquinone to aminohydroquinone (Figure 4B).

Aminohydroquinone is extremely unstable (Schenzle et al., 1997; Yin and Zhou, 2010); therefore, its structure analogs chlorohydroquinone (CHQ) and hydroquinone (HQ) were used to identify the ring-cleavage function of MnpC. H_6_-MnpC catalyzed rapid degradation of both CHQ and HQ, together with the accumulation of respective product with a λ_max_ of 320 nm (Figure S3). In contrast, neither substrate consumption nor product accumulation was observed when His_6_-MnpC was omitted from the reaction mixtures. Moreover, *mnpC* was co-transcribed with *mnpA* in 2C5NP-induced strain CNP-8. Therefore, MnpC was likely involved in the ring-cleavage reaction of 2C5NP degradation. So far, two kinds of (chloro)hydroquinone dioxygenase were reported. The linE-like single-subunit dioxygenases were reported to split the ring of CHQ between C1 and C2 (Miyauchi et al., 1999; Ohtsubo et al., 1999), whereas the HapCD-like two-subunit dioxygenase catalyzed the ring cleavage of CHQ between C1 and C6 (Moonen et al., 2008; Min et al., 2014). MnpC, a single-subunit aminohydroquinone dioxygenases reported here exhibits moderate identity (42%) with LinE, but has no sequence homology to HapCD. Therefore, the ring-cleavage position of aminohydroquinone catalyzed by MnpC during 2C5NP degradation was proposed between C1 and C2 with formation of 6-amino-4-hydroxy-6-oxohexa-2,4-dienoic acid (Figure 4B).

### *mnpA* is essential for 2C5NP catabolism in strain CNP-8

**Figure 8:**
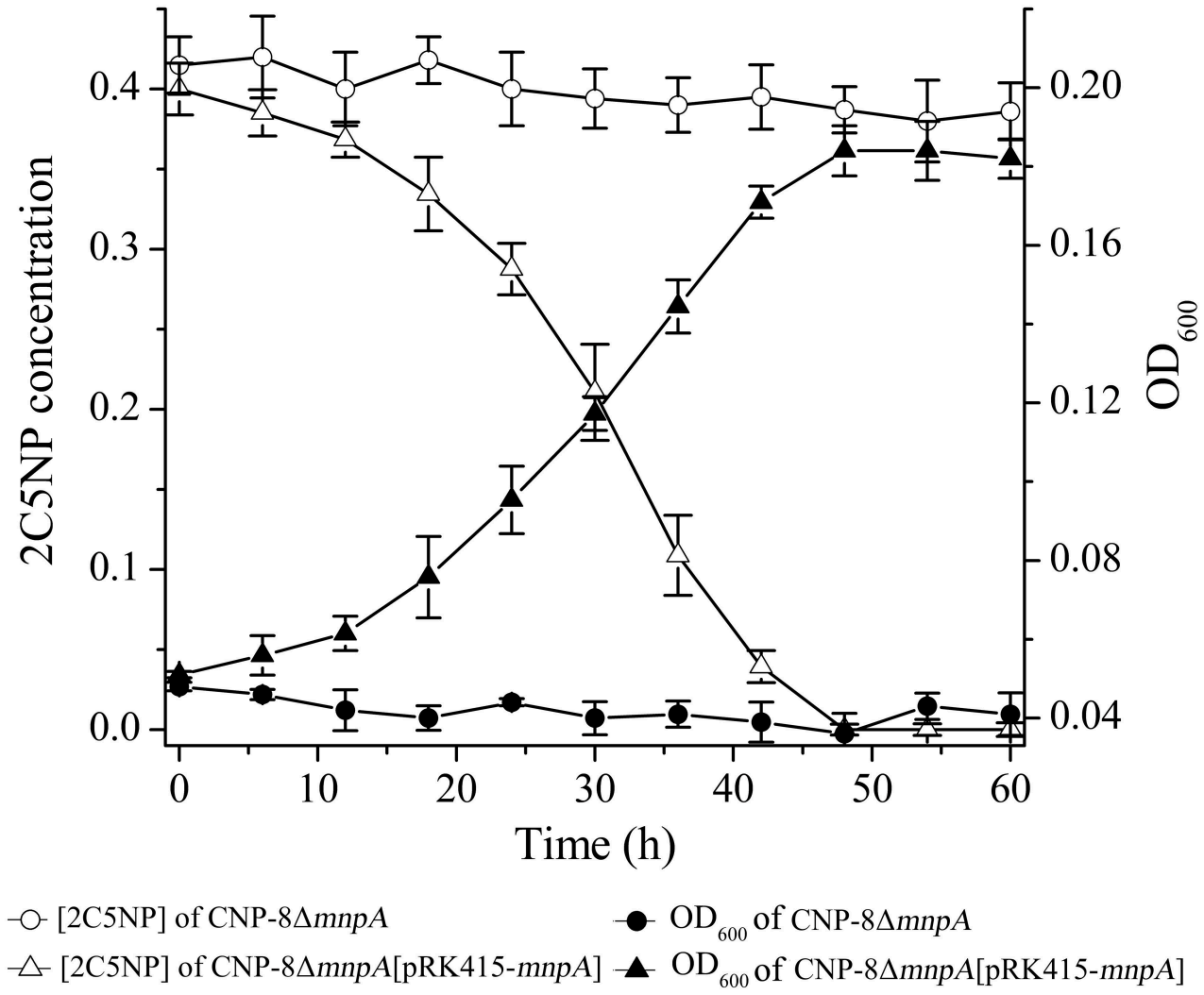
Time course of 2C5NP degradation and cell growth of *Cupriavidus* sp. strains CNP-8Δ*mnpA* (with *mnpA* deleted) and CNP-8Δ*mnpA*[pRK415-*mnpA*] (with *mnpA* complemented). The results of MNP are similar to 2C5NP, and they were not shown. All experiments were performed in triplicate, and error bars indicate standard deviations.

To determine the involvement of MnpA in 2C5NP catabolism *in vivo*, a mutant of strain CNP-8 with substitution of *mnpA* by a kanamycin resistant gene *nptII* was constructed through homologous recombination. Functional analysis showed that strain CNP-8Δ*mnpA* (with *mnpA1* deleted) was no longer able to utilize 2C5NP as well as MNP (Figure 8). This indicated that *mnpA* is necessary for strain CNP-8 to grow on both 2C5NP and MNP. Although the *mnpA1*-complemented strain CNP-8Δ*mnpA*[pRK415-*mnpA*] regained the ability to utilize these two nitrophenols, it exhibited lower maximum degradation rate compared to the wild-type strain CNP-8 (Table 2).

## Conclusion

*Cupriavidus* sp. strain CNP-8, the second bacterium with the ability to utilize 2C5NP, was isolated from pesticide-contaminated soil. Succinate was proved as the best additional carbon source during 2C5NP degradation by strain CNP-8. Biodegradation assays indicated that this strain is a potential and efficient candidate for biotreatment of 2C5NP-containing industrial effluents. MnpA catalyzes the partial reduction of 2C5NP to 2-chloro-5-hydroxylaminophenol via 2-chloro-5-nitrosophenol which was firstly identified during 2C5NP catabolism, and its encoding gene is necessary for strain CNP-8 to utilize 2C5NP. MnpC is likely responsible for the ring-cleavage reaction of 2C5NP degradation. This study fills a gap in the knoledge of the molecular mechanism of microbial 2C5NP degradation.

## Author contributions

JM and XH designed the experiment, JM and JW performed the experiment, JM and WC analyzed data, JM and XH wrote the paper.

### Conflict of interest statement

The authors declare that the research was conducted in the absence of any commercial or financial relationships that could be construed as a potential conflict of interest.
