# Supplementary material for: Genetic and Biochemical Characterization of 2-Chloro-5-Nitrophenol Degradation in a Newly Isolated Bacterium, Cupriavidus sp. Strain CNP-8
Source: Front Microbiol. 2017 Sep 13;8:1778. doi: 10.3389/fmicb.2017.01778 (PMC5604080; doi:10.3389/fmicb.2017.01778)
Supplement: Supplementary file 1 [file DataSheet1.PDF]

Supplemental files

Genetic and Biochemical Characterization of 2-Chloro-5-nitrophenol degradation in a newly isolated bacterium, *Cupriavidus* sp. strain CNP-8

Jun Min[1], Weiwei Chen[1], Jinpei Wang[2] and Xiaoke Hu[1*]

[1] Key Laboratory of Coastal Biology and Bioresource Utilization, Yantai Institute of Coastal Zone Research, Chinese Academy of Sciences, Yantai 264003, China

[2] Key Laboratory of Agricultural and Environmental Microbiology, Wuhan Institute of Virology, Chinese Academy of Sciences, Wuhan 430071, China

[*]Corresponding author: Xiaoke Hu, Email: xkhu@yic.ac.cn; Tel: 86-535-2109127

Table S1 Primers used in this study

Primers	Sequence(5'-3')*	Purpose or reference
27-F	AGAGTTTGATCMTGGCTCAG	(Polz and Cavanaugh 1998)
1492-R	TACGGYTACCTTGTTACGACTT	
RTq16S-F	CGTGTAGCAGTGAAATGCGTAGAG	To amplify a 142 bp fragment
RTq16S-R	GACATCGTTTAGGGCGTGGAC	of 16S rDNA for RT-qPCR
RTq-*mnpA*-F	GATGGACCGCCGCCTGGAATG	To amplify a 136 bp fragment
RTq-*mnpA*-R	GCGTAGCACGGGATACTGGAGC	of *mnpA* for RT-qPCR
RTq-*mnpB*-F	GATTTCCGTTTCACCGATACC	To amplify a 142 bp fragment
RTq-*mnpB*-R	TGTCCGAAGCCTCGATACC	of *mnpB* for RT-qPCR
RTq-*mnpC*-F	CACCACCGCTTTGAGACTGCC	To amplify a 161 bp fragment
RTq-*mnpC*-R	CGCTCCTTGAGGACCATCTGC	of *mnpC* for RT-qPCR
RTq-*mnpD*-F	GGCAACCAGTTCCGCATCGTC	To amplify a 139 bp fragment
RTq-*mnpD*-R	GCTGCTCTACGGCTTTCATCCC	of *mnpD* for RT-qPCR
mnpA-F	AGGAGACATATGAACGATGCCCTGATCACCG	To amplify *mnpA* gene
mnpA-R	GGCCTCGAGTCAGGCTTCGACGATGGAAAC	for expression
mnpB-F	AGGAGACATATGGCCCAAAGCGTTGCAGATG	To amplify *mnpB* gene
mnpB-R	GGCCTCGAGTTACAGCGAGTAATACATTTCGAATTC	for expression
mnpC-F	AGGAGACATATGTCGATGATCAAAGGATTTCATC	To amplify *mnpC* gene
mnpC-R	GGCCTCGAGTTACGCGACGATCGGCTCAAG	for expression
mnpD-F	AGGAGACATATGAGCAGTGATCAATTGATGAACC	To amplify *mnpD* gene
mnpD-R	GGCCTCGAGCTACGCCGGTGCCACCTCGGC	for expression
GC-*mnpA*-F	GACAAGCTTATGAACGATGCCCTGATCACCGGG	To amplify *mnpA* for
GC-*mnpA*-R	GACGGTACCTCAGGCTTCGACGATGGAAAC	gene complemetation
KO-mnpA$_u$-F	CCATGATTACGAATTATCCGGGAATGGCGGCAATACC	To amplify upstream fragment
KO-mnpA$_u$-R	AGAGATTTTGAGACACTTTGTGTCTCCAGTGATGGCTCTT	of *mnpA* for gene knockout
KO-mnpA$_d$-F	GATGAGTTTTTCTAATTGGTCTGGCTTCCGGGTCTGGC	To amplify downstream fragment
KO-mnpA$_d$-R	GGCCAGTGCCAAGCTCACCGCCCGGCATCTTGAAATAC	of *mnpA* for gene knockout
KO-kan-F	TGTCTCAAAATCTCTGATGTTAC	To amplify kanamycin resistance
KO-kan-R	TTAGAAAAACTCATCGAGCATC	gene *nptII* for gene knockout

* Specified restriction sites are underlined.

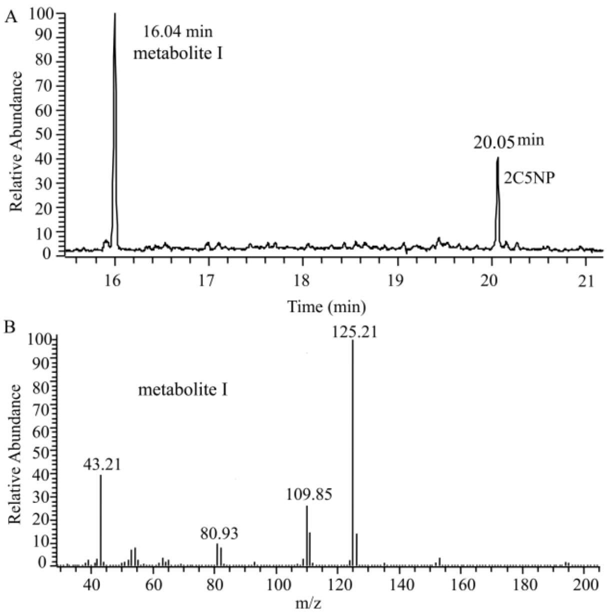

Fig. 1 GC-MS analysis of the intermediate captured during 2C5NP degradation under anaerobic condition with 2C5NP-induced strain CNP-8. (A) The gas chromatogram is the extracted ion current chromatogram at m/z 125.00±0.50 and 173.00±0.50 from the total ion current chromatogram. (B) Mass spectra of metabolite I.

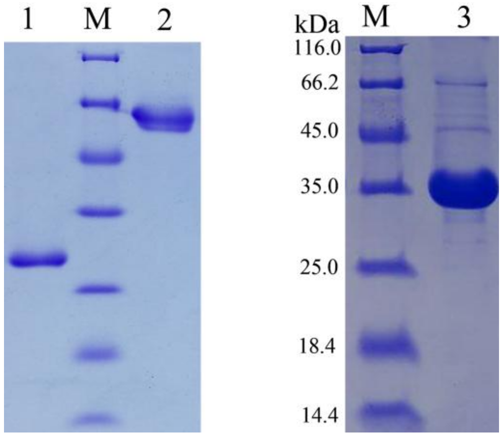

Fig. S2 SDS-PAGE of purified recombinant Mnp proteins. Lane M: molecular mass standards; lane 1: purified His_6-MnpA; lane 2: His_6-MnpB; lane 3: His_6-MnpC.

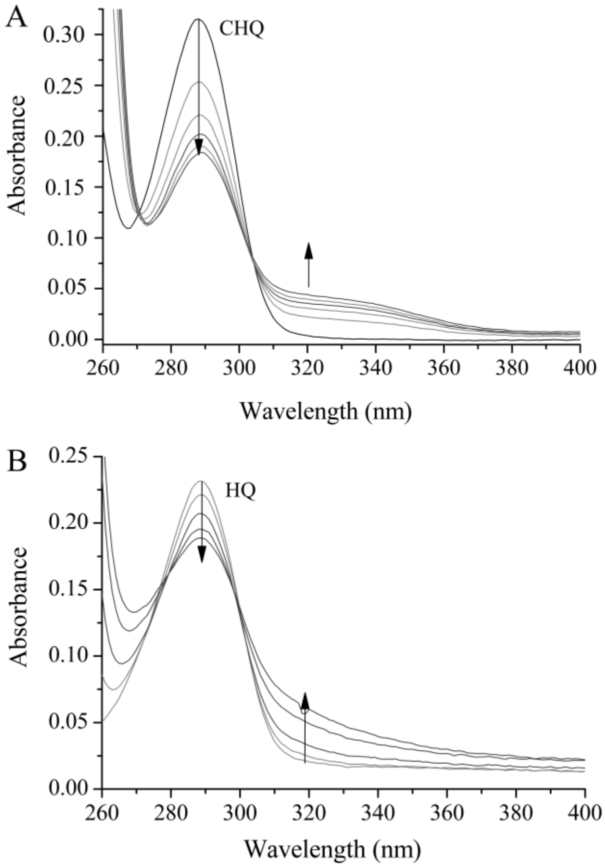

Fig. S3 Spectral changes during the conversion of CHQ (A) and HQ (B) catalyzed by purified H_6-PnpC. The spectra were recorded every minute after the addition of substrates. The arrows indicate the directions of spectral changes.
